# Embroidered Interdigitated Electrodes (IDTs) with Wireless Readout for Continuous Biomarker Monitoring

**DOI:** 10.3390/s24144643

**Published:** 2024-07-17

**Authors:** Emmy L. Amers, Bethany V. Orme, Yuyuan Shi, Hamdi Torun, Linzi E. Dodd

**Affiliations:** 1 Smart Materials & Surfaces Laboratory—E-Textiles Centre, Department of Mathematics, Physics and Electrical Engineering, Northumbria University, Newcastle upon Tyne NE1 8ST, UKhamdi.torun@northumbria.ac.uk (H.T.); 2 Digital Textile Lab, School of Design, Faculty of Arts, Design and Social Sciences, Northumbria University, Newcastle upon Tyne NE1 8ST, UK

**Keywords:** wearable technologies, health monitoring, interdigitated electrodes, embroidered sensors, glucose sensor

## Abstract

Non-invasive continuous health monitoring has become feasible with the advancement of biosensors. While monitoring certain biomarkers such as heart rate or skin temperature are now at a certain maturity, monitoring molecular biomarkers is still challenging. Progress has been shown in sampling, measurement, and interpretation of data toward non-invasive molecular sensors that can be integrated into daily wearable items. Toward this goal, this paper explores the potential of embroidered interdigitated transducer (IDT)-based sensors for non-invasive, continuous monitoring of human biomarkers, particularly glucose levels, in human sweat. The study employs innovative embroidery techniques to create flexible fabric-based sensors with gold-coated IDTs. In controlled experiments, we have shown the variation of glucose concentration in water can be wirelessly detected by tracking the resonant frequency of the embroidered sensors. The current sensors operate at 1.8 GHz to 2 GHz and respond to the change in glucose concentration with a sensitivity of 0.17 MHz/(mg/dL). The embroidered IDT-based sensors with wireless sensing will be a new measurement modality for molecular wearable sensors. The establishment of a wireless sensing mechanism for embroidered IDT-based sensors will be followed by an investigation of sweat for molecular detection. This will require adding functionalities for sampling and interpretation of acquired data. We envisage the embroidered IDT-based sensors offer a unique approach for seamless integration into clothing, paving the way for personalised, continuous health data capture.

## 1. Introduction

Wearable technologies have seen significant advances and development in recent years for health and well-being purposes enabling the creation of personalised medicine and assistance platforms [1]. Using wearable technologies for individual health monitoring has garnered the attention of not only clinicians but that of the wider public who want to have access to increased knowledge and awareness of their own medical status. An important enabler for these possibilities is the capability of human biomarkers to be detected more effectively and with as little impact on the individual as possible [2]. Continuous biomarker monitoring is known for providing significant insight into the long-term and current health status of an individual [3,4]. Technologies focused on biomarkers have been developed to gather and process data, monitor actions, and then tailor the experiences to meet the needs of the users depending on the obtained and recorded data [5]. The current value of the global wearable technologies market is USD 61.30 billion and is expected to grow exponentially at a compound annual growth rate (CAGR) of 14.6% from 2023 to 2030 [6].

The vital signs of an individual are one of the most significant measures of the health of a human. A change in heart rate, temperature, or respiration rate can signify a potentially significant health issue and these are therefore carefully monitored in point-of-care (POC) environments [7]. However, as people become ever more health conscious, the requirement to be able to measure these signs in a day-to-day manner has increased leading to the introduction of smart monitoring systems that not only measure these vital signs but also track any changes over time [8]. Early iterations of this technology were primarily used within physical activity monitoring. Their usage has gained popularity and their functionality has improved as increased focus on research and development has been directed into this area [9]. These technologies have grown in accessibility and affordability in recent times and their functionality variability has progressed since their introduction to the health monitoring markets. Smaller and more sensitive components have made it feasible to create more compact accessories, which were before harder if not impossible to achieve [5].

Whilst these vital signs are relatively easy to measure, they provide little information on the underlying cause for an elevation, reduction, or overall tracked change in these levels, meaning that further investigation is always required to identify the root cause. This either involves taking scans of the body (X-ray, CT, MRI, etc.) or samples of bodily fluids with blood samples being the most common. Blood samples can provide additional information on blood glucose levels, cholesterol, and inflammation [10] markers, all of which are identifiable by the levels of biomarkers present in the sample [1,11]. As blood tests require an invasive sampling method, these tend to have to be performed in a healthcare setting with the sample being sent to laboratories for the specific testing required. This means that the cold chain movement of the samples can be required, increasing the overall costs of performing the tests as well as a sometimes significant waiting time for results to reach the patient. Blood glucose measurements are an exception; however, as devices that both take a sample and read the glucose level have been developed and are widely used by patients in their own homes.

Biomarkers can also be identified in other forms of bio-fluids such as saliva, urine, and sweat—the collection of which are classified as non-invasive methodologies for obtaining a sample [2]. Non-invasive methods have their advantages as they address the issue of patient comfort; however, the types of biomarkers present within the sample type vary and are generally at a lower level than the biomarkers present in blood samples, requiring tests with lower detection limits [12] or a higher volume sample [13]. Sample contamination is another problem that these sample types encounter as they are more susceptible to contact with external contaminants. Salivary samples are generally contaminated by food or drink consumed by the individual but the samples can also be contaminated by the prior cleaning procedure with things such as mouthwash [14]. Sweat can be obtained more readily from a range of non-invasive ways at areas of high concentrations of sweat glands or via iontophoresis [15] and is suitable for continuous monitoring [2,16,17] but is still susceptible to contamination via biofouling [18]. In 2012, Moyer et al. [19] performed a study that showed a correlation between glucose measurements taken from capillary blood and those measured in sweat. Sweat sensing has also been effectively demonstrated using electrochemical sensors typically operating in amperometric, potentiometric, and voltammetric modalities using electrodes and via electrical connections to the electrodes [20]. The electrodes can be in various form factors, and an effective way to increase the sensitive area of an electrode is by using interdigitated electrodes. Devices using these electrodes are called interdigitated transducers (IDTs), which enable various detection modalities, including capacitive and acousto-mechanical sensing techniques [21,22].

In order for the measurement of biomarkers to become part of everyday life, tracking the change in the levels of biomarkers has to be conducted in a continuous manner, which inevitably requires the use of a non-invasive technique, which ideally could be performed with little input from the user [1,2,15]. Wearable technologies have long been used in the form of smartwatches and other devices to monitor vital signs and have recently expanded to include invasive measurement of insulin for diabetic patients. The first wearable sensor designed to measure a biomarker was an electrochemical tattoo for lactate levels, capable of performing real-time measurements [23]. However, the sensor surface required functionalisation to be specifically sensitive to this biomarker. The method of functionalisation depends on the specific biomarker that is being targeted and can therefore make a sensor specific to a certain measurement.

The breadth of modalities and form factors for wearable devices has been increasing including the emergence of fabric-based sensors as potential progression pathways for wearable technologies in the future and reducing some of the barriers to implementation [17,24]. Embroidered sensors could be advantageous as this would allow for seamless integration into any item of clothing, offering a unique approach to capturing personal data without disrupting a person’s usual routine [25,26]. Thread-based multiplexing sweat sensors [27], fibre [28], and gold woven sensors [29] have been shown to detect targeted biomarkers, with each method providing two different methodologies to apply the sensing material to fabrics and clothing.

The requirement for these measurements to be performed wirelessly and to seamlessly integrate with current technologies is an increasing demand [15]. Wireless sensing opportunities have been demonstrated with IDTs coupled to external antenna structures [30,31]; however, the reading and transfer of data can require large items of equipment, which is unsuitable for all application spaces. In extreme environments—such as space, deserts, or deep underwater—the tracking of an individual’s overall health becomes critically important but increasingly challenging. Environmental factors such as extreme cold, immense hyperbaric pressure, or submersion in water not only pose challenges for effective measurements [32] but can also create communication issues between the measurement device and the system processing the data.

In this work, embroidered fabric-based IDT sensors were investigated for droplet-sensing applications. The sensors were manufactured using unique embroidery techniques with various thread combinations, and the subsequent IDT frequency response was monitored with an external antenna for non-contact wireless measurements. Changes in frequency were measured in the presence of pure deionised (DI) water and at increasing glucose concentrations, providing a feasible path for detecting glucose present in human sweat. The results demonstrate the effectiveness of a wireless sensing mechanism for embroidered IDT-based sensors. The structures have been systematically investigated for their coupling with external antennas in measuring liquid droplets. These investigations will be followed by the development of added functionalities for sampling and data interpretation toward a sweat analysis platform.

## 2. Materials and Methods

**Figure 1:**
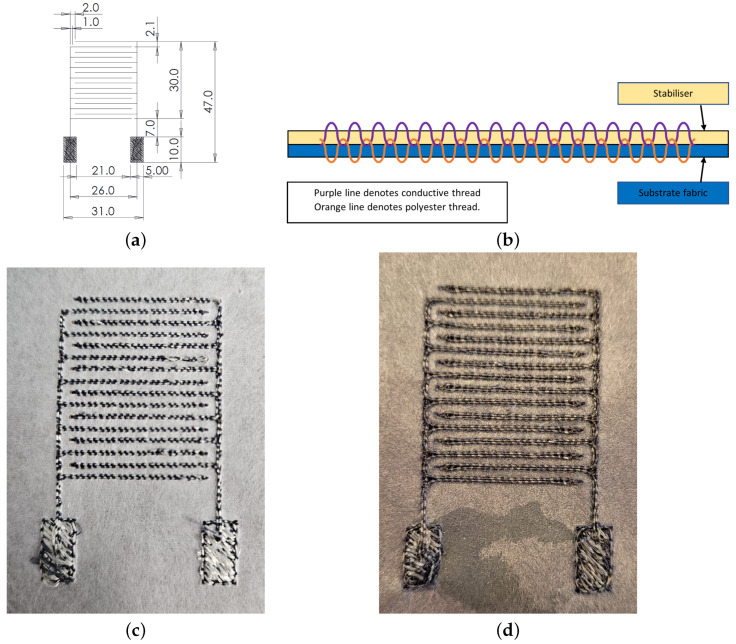
Fabrication of embroidered IDTs. (**a**) Embroidered IDT design and stitch pattern (dimensions in mm). (**b**) The schematic cross-section of the embroidery process. (**c**) Embroidered IDT (silver thread) prior to gold metal deposition. (**d**) Embroidered IDT (silver thread) after gold metal deposition, and prior to the removal of the tearaway stabiliser, which acts as a shadow mask.

To produce a flexible fabric-based sensor, an IDT design was embroidered into Calico fabric (Brother Entrepreneur Pro-X PR1050X, Brother International Europe Ltd., Manchester, UK). The design of the IDT was created using PE-Design 11 software, using running stitches with a stitch density of 0.5 stitches/mm to 1 stitches/mm. The IDT fabrication consisted of two different types of thread: a standard Brother polyester thread (135 dtex/2), which was wound onto the top spool, and a silver-coated liquid crystal polymer fibre (Liberator Kuraray Vectran), which was run through the bottom bobbin, resulting in an inverted embroidery process, as shown in Figure 1. Having the threads in this configuration allows for varying thread tensions and reduces the number of contact points for the relatively inelastic conductive thread. The Calico was made inflexible through the embroidery process by using a tearaway stabilizer to ensure minimal distortion of the design. Post embroidery, the silver thread must be coated with a layer of gold to provide the correct structure onto which specific sensing chemicals can be attached; see Figure 1d. 100 nm of gold was thermally evaporated at a base pressure of 1 × 10^−6^ mbar in a Leybold UNIVEX 250 thermal evaporation system (Leybold GmbH, Cologne, Germany). Gold was purchased from Testbourne Ltd., Hampshire, UK, at a purity of 99.99%. The tearaway stabiliser acted as a shadow mask, ensuring that only the silver-threaded area and not the Calico base was coated. The stabiliser could then be easily removed from the sensor for testing, which left the conductive silver thread coated with 100 nm of gold, and the substrate remained uncoated.

**Figure 2:**
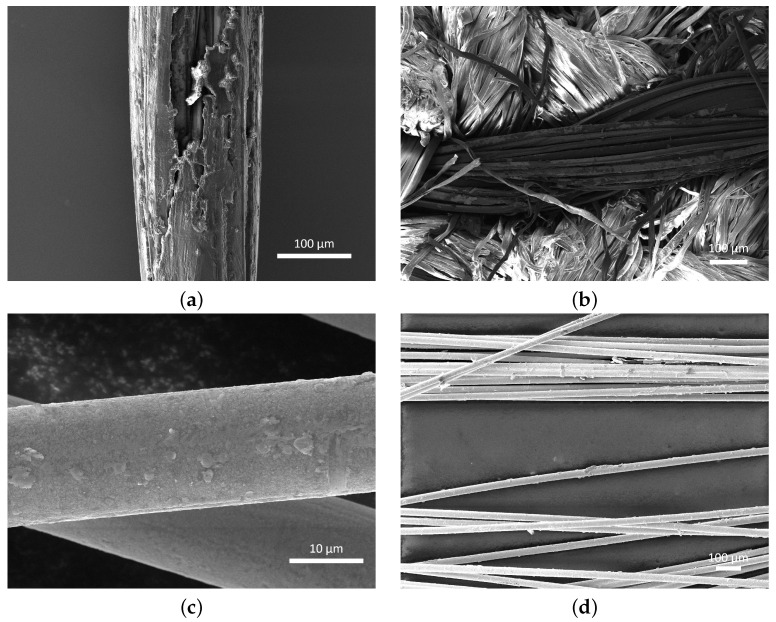
SEM images comparing Swicofil SwicoGold threads and the Syscom Liberator silver thread. (**a**) SEM image of a SwicoGold fibre prior to embroidery (Mag: 587×). (**b**) SEM image of the SwicoGold thread after embroidered into the Calico fabric (Mag: 293×). (**c**) SEM image of a silver fibre (Mag: 10.5k×). (**d**) SEM image of multiple silver-coated fibres, which twist together to form the embroidered thread (Mag: 349×).

Pre-coated gold thread (SwicoGold, Swicofil AG, Emmenbrücke, Switzerland) was also investigated as a thread option to eliminate the need for post-gold coating. Figure 2a shows an SEM image of a SwicoGold thread fibre with an average diameter of 200 µm, with the gold coated on top of a polyethylene terephthalate (also known as polyester) fully drawn yarn (PET FDY) base. In the centre of the image, it can be seen that the gold coating is not uniform, with an area of exposed polymer visible. This could be due to the coating process or the fragile nature of the coating. After undergoing embroidery, further damage has occurred to the gold coating of the thread (Figure 2b), with larger areas of uncoated fibres appearing throughout the thread, leaving a non-uniform gold sensing surface, which would subsequently require an additional coating of gold to obtain a uniform coating.

Comparing the SEM image of the single fibre of Swicofil SwicoGold thread with a single fibre of Syscom Advanced Materials Liberator silver (Figure 2c), the differences in metal coating uniformity can be seen with the silver uniformly coating the underlying polymer fibre, with very few areas of damage evident, but some roughness present. Expanding this analysis to multiple fibres within the thread (Figure 2d), the evidence of coating roughness increases; however, the coating again appears to be fairly uniform with no identifiable areas of coating damage. This surface provides a uniform metal base onto which the gold can be deposited, creating an overall uniform layer of gold for the subsequent sensing chemical attachment.

**Figure 3:**
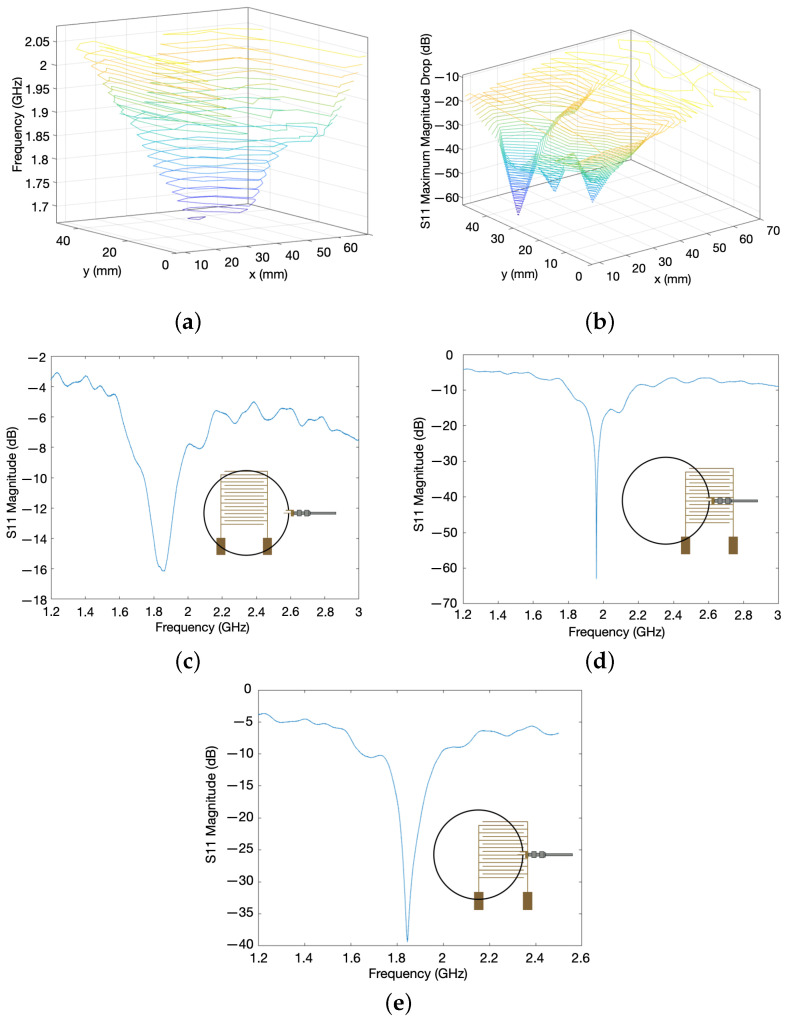
Method and experimental setup for fabric-based embroidered IDT testing. The process of selecting the preferred relative positions of the hoop antenna and IDT are shown, with a compromised position chosen to give a satisfactory response with a significant overlap. (**a**) The effects of the relative position of the loop antenna and IDT on the frequency of the resonance dip for IDT 1. (**b**) Diagram of the full antenna coverage and associated frequency response. (**c**) Diagram of the full antenna coverage and associated frequency response. (**d**) Diagram of the optimum relative position of IDT, antenna, and associated frequency response. (**e**) Diagram of compromised antenna position relative to IDT to have good coverage, significant overlap, and associated frequency response.

Figure 1 shows the design parameters of the IDT and the method of embroidery on a fabric substrate. The structures are transferred on the fabric substrate using a pair of conductive and polyester threads with the help of a stabiliser, as shown in Figure 1b. The frequency response of the IDT sensor was measured using a Vector Network Analyser (VNA: Agilent Technologies E8364B, Agilent Technologies, Santa Clara, CA, USA). The sensor was placed onto a grid to allow the exact position of the sensor in relation to a fixed copper loop antenna to be measured and controlled. The copper loop antenna (thickness 1.5 mm, diameter 48 mm) was placed at an average fixed distance of 2 mm above the IDT, ensuring that no stray fibres from the sensor contacted the antenna. The VNA was set to perform 3201 sweep points across a frequency range of 1.2 GHz to 2.5 GHz. Specifically, the input reflection coefficient measured at the first port, S11, and at the second port, S22, were monitored to measure the resonant frequency of the antenna–sensor coupled system. The sensor was moved to produce a resonant frequency response map (Figure 3a), indicating that along the legs of the IDT, which connect the IDT digits to the pads, the frequency is highest at 2.09 GHz, and along the middle of the IDT where the overlap of the digits is furthest away from either the left or right leg, the lowest region of frequency is seen at 1.66 GHz. By plotting the change in position as a function of the magnitude of the resonant peak (Figure 3b), it can now be seen that the area of the highest magnitude is located along the left leg of the IDT, matching one of the areas of highest frequency. The magnitude then decreases when moving along the x-direction toward the right-hand leg. A further area of high magnitude is not seen along the right-hand leg due to the orientation of the antenna used (Figure 3c inset).

To achieve a maximum detection region, the sensor should be placed at the most central location, incorporating both the digits and the pads at x = 42.0 mm and y = 35.0 mm (Figure 3c); this however only provided a resonant frequency magnitude dip of −16.2 dB. The optimum magnitude dips of −63.0 dB are located at position x = 14.0 mm and y = 35.0 mm, providing very little coverage of the sensor, minimising the detection region (Figure 3d). In order to optimise both the magnitude dip and the detection region, a compromise is made with the measurement positions at x = 28.0 mm and y = 28.0 mm, bringing the antenna further up from the pads and centring it with the left-hand leg, resulting in a magnitude dip of −39.4 dB (Figure 3e).

If the sensor was inverted with the gold IDT facing away from the antenna, the magnitude of the significant resonance dip was reduced from −39.4 dB to −29.9 dB but was still located in the same area; thus, the sensor could be used in either orientation. The resonance dip was also seen in the same location across sensors with the same design parameters. All subsequent experiments took place with the conductive side of the IDT facing upwards.

## 3. Results and Discussion

It was previously demonstrated that IDT-based structures can be coupled with interrogating antennas to excite the IDTs electromagnetically. For example, IDTs in circular [31] and rectangular [30] geometries were coupled with loop antennas for sensing applications. Specific resonances can be induced when the excitation conditions help induce the circulating current across the IDTs or the fingers are electrically polarised. The resonances can be observed by measuring the frequency response of the interrogating antenna. Specifically, for a loop antenna, the resultant resonant frequencies arising due to the coupling of the antenna with the IDT can be observed on the S11 spectrum. The resonant frequency depends on the geometry of the IDT as well as the effective capacitance and the inductance imposed by the environmental parameters.

**Figure 4:**
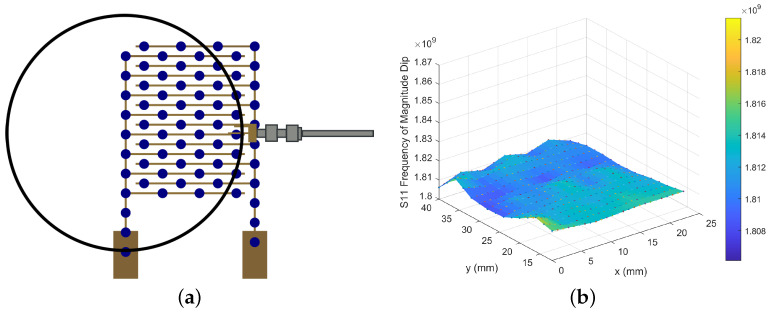
Measurement position of DI water droplets and the corresponding frequency response of the waterproofed sensor. (**a**) Measurement positions of water droplets on the IDT. (**b**) Water droplets are dispensed onto the tape covering the IDT (frequencies reported in Hz).

To first examine the most sensitive location over the surface of the sensor, the surface of the sensor was covered with heat-resistant tape (Kapton tape) to provide a temporary waterproofing layer and 10 μL droplets of DI water (18 MΩcm Millipore) were dispensed onto the covered surface of the IDT sensor, in positions detailed in Figure 4a. S11 spectra of the antenna–sensor coupled structure were measured while the droplets were in each set stationary position over the surface, as shown in Figure 4b. The Kapton tape and DI water droplets introduced dielectric loading onto the IDT, increasing the effective capacitance and, thus, decreasing the resonant frequency of the antenna–sensor-coupled structure. Once the position of the antenna was fixed, the range of positions for droplet placement was reduced, resulting in a new x-position range of 0 mm to 20 mm and a y-position range of 0 mm to 12 mm.

**Figure 5:**
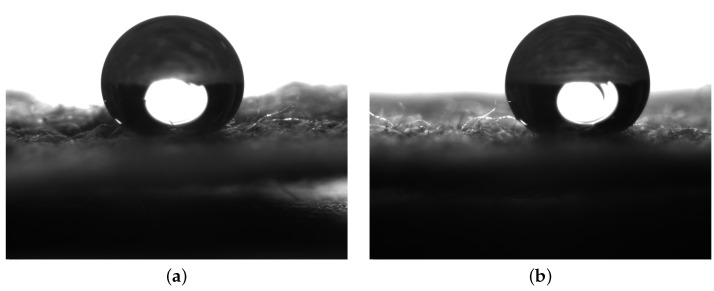
Static contact angle measurements performed on IDT sensors pre- and post-Kapton tape application; (**a**) 10 μL DI water droplet deposited onto the IDT sensor prior to any tape being placed onto the surface; (**b**) 10 μL DI water droplet deposited onto the IDT sensor after the Kapton tape had been removed from the surface.

The Kapton tape waterproofing layer was removed from the sensor surface, exposing the IDTs, and the single droplet experiment was repeated. When droplets were placed onto the sensor, a high static water contact angle of approximately 140° was observed. Static contact angle measurements were carried out using a Krüss DSA 30 on the sensor prior to the Kapton tape being applied, and after removal, to ensure that the measured angle was not caused by the residue of adhesive from the tape. Moreover, 10 μL droplets were placed onto the surface; however, due to the hydrophobic nature of the surface, multiple deposition attempts were required in the same area before the droplet detached from the 0.7 mm diameter needle. Figure 5 shows two example droplet images on the surface before tape application (Figure 5a) and after tape removal (Figure 5b). In both images, the large contact angle is evident, but the contact points are obscured by the uneven nature of the fabric-based sensor surface. ImageJ was used to enhance the brightness and contrast of the two images to highlight the contact points and the baseline of the droplet before using the built-in angle tool to measure the static contact angle. The contact angle measured before the addition of tape was measured to be 151° ± 1° and post-tape application and removal 149° ± 1°. This indicates that the tape does not leave a residue affecting the wettability of the surface, and the observed hydrophobic nature is an inherent property of the fabric and thread used to create the sensor.

**Figure 6:**
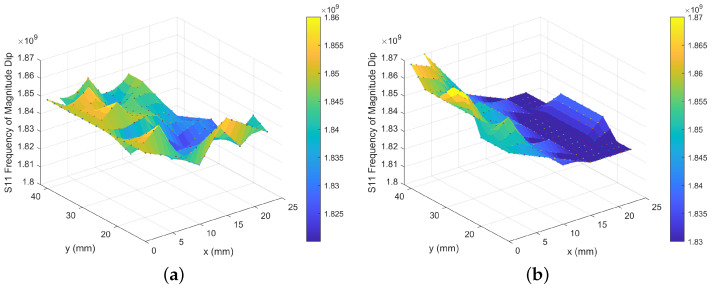
Detection of water on IDTs for different droplet application methods at different positions on the IDTs (all frequencies reported in Hz). (**a**) Individual water droplets are deposited directly onto the IDT, with IDT dried between each droplet. (**b**) Detection of water on IDTs for different droplet application methods at different positions on the IDTs (all frequencies reported in Hz).

Because the static contact angle is large, there is very little water in direct contact with the surface, allowing the droplet to be easily removed by an absorbent material. To ensure the droplet had not wetted the sensor, weight measurements before and after droplet placement were performed using a precision balance (Denver Instrument TP-214, Denver Instrument Company, Denver, CO, USA); these confirmed that the entirety of the droplet was removed from the surface so the sensor did not require drying prior to the next droplet measurement. From the frequency shift data of the single droplets (Figure 6a), an area of higher frequency can be seen along y for x = 0 mm to 5 mm, which is the same position along the left-hand leg of the IDT where the significant magnitude dip in Figure 3b and high frequencies of Figure 3a were also located. The frequency measured along this left-hand leg is equivalent to the frequencies measured with no object on the surface (Figure 3e); however, the frequency shifts and reduces as the droplets are placed at positions further away from this left-hand side (x = 5 mm to 20 mm), giving a maximum reduction of 0.04 GHz from a starting frequency of 1.81 GHz to 1.85 GHz.

Using the droplet placing system detailed in Figure 4a, the droplet’s footprint covers only a single metallic line over the area of the IDT during measurements. Any water droplet bridging two metallic lines would alter the single resonant frequency dip in a way or introduce additional dips that would prevent tracking the frequency effectively.

Single droplet experiments allowed for the measurement of the sensor’s response in the areas where droplets were deposited. In order to measure the entire sensor’s response, a cumulative droplet experiment was performed.

To simulate the accumulation of fluid on the sensor, droplets were placed one after the other onto the IDT in the same positions, as shown in Figure 4a, starting from the top left-hand corner and working from left to right down the IDT fingers. Instead of removing the previous droplet before placing the next, the previous droplet was left in place on the surface. The initial ten droplets sat on the surface at a high static contact angle (CA approximately 140°). As the number of droplets increased, a breakthrough threshold was reached at an approximate total volume of 150 μL with the liquid eventually penetrating and wetting the fabric. Figure 6b details the frequency response measured, with the left-hand leg of the sensors exhibiting the highest frequency, as seen in Figure 6a, and the frequencies decreasing as the right-hand leg is approached. However, in the single droplet experiment (Figure 6a), there is a clear small area of the greatest frequency shift, dependent on both y and x measurement positions, whereas for the cumulative droplets (Figure 6b), the y position appears to have less of an effect on the frequencies measured and, hence, the greatest frequency shift can be seen by moving only in the x direction. Once the test was complete, the sensor was allowed to dry in ambient laboratory conditions (temperature: 20 °C to 25 °C, humidity: 35% to 50% RH).

**Figure 7:**
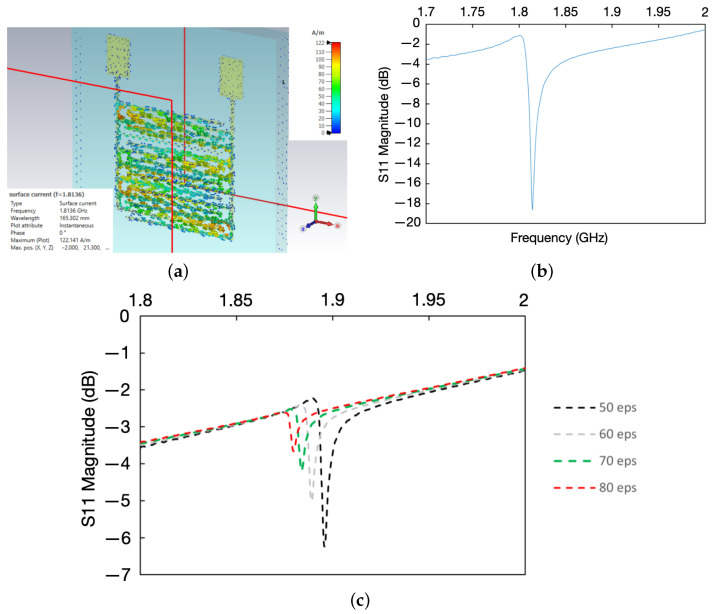
CST simulation of the IDTs, showing the difference in sensitivity in different regions, the resonant dip of the IDT pattern, and the effect of different epsilon values of droplets placed on the simulated IDT. (**a**) Simulation setup showing the areas of highest sensitivity. (**b**) Experimental resonant frequency dip of the IDT. (**c**) Comparison between the simulated and experimental resonant frequency dips.

The IDTs were also modelled using CST Microwave Studio Suite 2022, 3D EM analysis software, with the structures excited by plane waves to obtain their resonant frequencies (Figure 7a). The experimental resonant frequency dip of the sensor is shown in Figure 7b. A liquid droplet consisting of water with a fixed volume and varying permittivity was then placed on the IDT, at the previously described optimal location, to replicate the effects of different concentrations of glucose in sweat. This simulation revealed frequency shifts, with the resonant dip decreasing in frequency as permittivity increased, as shown in Figure 7c. Inevitable geometrical alterations due to the embroidery process, such as small defects between adjacent stitches, result in frequency differences between simulated and experimental values. However, the magnitude of the frequency shifts in both the simulations and experiments were comparable, validating the simulation model.

**Figure 8:**
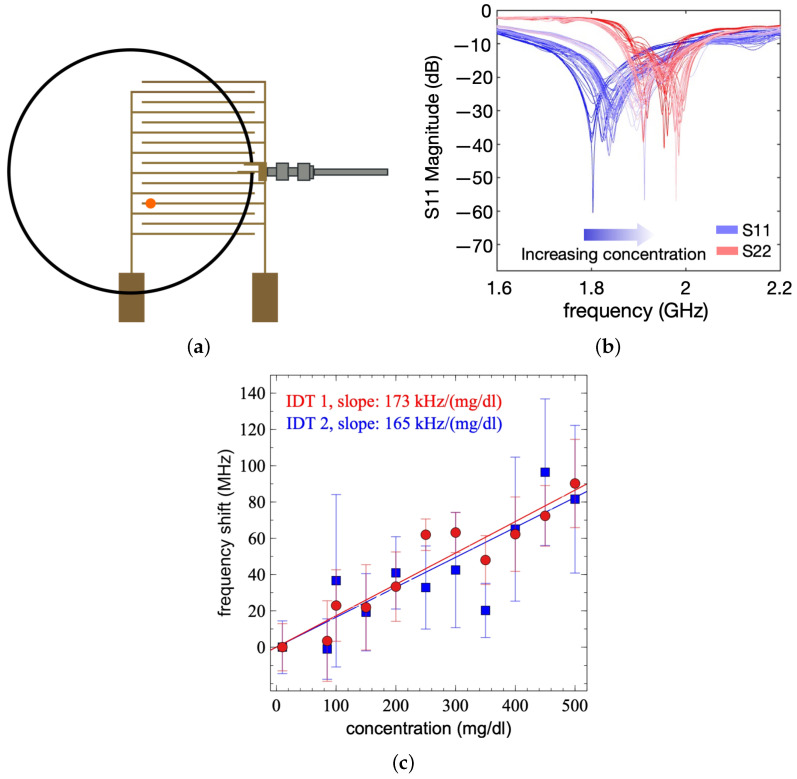
Glucose detection of the IDTs, including an optimal position for detection, the S11 and S22 traces for different glucose concentrations, and the effect of glucose concentration on the frequency change of the differential IDT measurement. (**a**) Measurement position of the glucose droplet in relation to the antenna and IDT. (**b**) S11 (measurement) and S22 (control) trace for glucose response. (**c**) Frequency shifts for different concentrations of glucose deposited at the optimal sensitivity location with differential measurements performed using DI water droplets on a separate IDT.

As a case study, droplets with various concentrations of glucose within the physiological range (10 mg/dL to 500 mg/dL) were used in subsequent experiments. The concentration range was selected to assess the sensor response within a range wider than the expected glucose concentration in the blood to assess the linearity. Samples were prepared by mixing DI water with glucose at various concentrations. Each droplet of glucose was placed onto the sensor in the highest sensitivity region (Figure 8a) and the frequency response was measured. A pair of IDTs were used in these experiments to compensate for the ambient variations that may affect the resonant frequency of the sensors. Specifically, the sensing IDT was connected to the first port. Droplets with varying concentrations of glucose were applied to the surface while S11 spectra were obtained. A control IDT was connected to the second port, and DI water droplets were placed on its surface while its S22 spectra were obtained.

As the static contact angles of glucose on the sensor were similar to those of pure water, the droplets could easily be removed from the sample. However, to ensure that no glucose remained on the surface, the measurement area was cleaned by placing five 10 μL DI water droplets onto the same area. By placing these droplets in quick succession onto the same position, the wetting breakthrough limit of the surface was reached after the third droplet; therefore, the surface required a drying step after the deposition of the five DI water-cleaning droplets. A hot air gun (Aouye Int 852A+) was used to quickly dry the sensor with the flow rate set to 99 (383 mL/s) and temperature to 100 °C. A brief time period was allotted for the cooling of the sensor and the antenna before the next measurement was performed. The sensor and antenna were determined to be ready for the next measurement when the measured frequency returned to the starting resonant frequency, this period of time was dependent on the ambient conditions at the time of measurement. The frequency measurements for the glucose droplets were performed by taking readings on S11, while S22 was used as the control, with DI water droplets placed in the same position. As ambient conditions such as temperature and relative humidity were not controlled throughout these experiments, this accounted for any changes to the resonant frequencies and, therefore, measured frequencies caused by external factors. As can be seen in Figure 8b, there has been a shift in the frequencies measured on S22, indicating that the external conditions have affected the experimental parameters. By using both S11 and the control S22, a normalised change in frequency can be calculated. This protocol was followed for two pairs of IDTs, and similar responses were obtained, as shown in Figure 8c). The measured frequency shift increases with the increasing glucose concentration, with a slope of 0.17 MHz/(mg/dL) (Figure 8c). For each concentration, the measurements were repeated five times, and the error bars in Figure 8c show the standard deviation of the measured frequencies. The increasing frequency shift with concentration indicates that the effective capacitance of the device is decreasing, consistent with the literature [31,33]. The sensitivity value that we report and how it compares with similar wireless resonator-based sensors are shown in Table 1. Unlike most similar sensors based on ring resonators, the basic structure of our sensor is an IDT. We have demonstrated that IDTs can be utilised as wireless resonant sensors when coupled with an interrogating antenna. Furthermore, these structures can serve as capacitive sensors when they are directly connected to conventional electrical circuits, such as oscillators, for readout purposes.

The experimental results show that the concentration of glucose can be detected wirelessly in water using embroidered structures and the readout antenna. The experiments were performed within physiologically relevant concentration ranges using two different pairs of embroidered structures with similar responsivity values. The obtained results are promising for a sensor that can be further improved, including better controls to reduce measurement uncertainties. It is important to note that the experiments were conducted with droplets introduced onto the surface of the structures without any specific controls, and the antenna was coupled to the structures without any housing or mechanism for precise registration. Relatively relaxed conditions were deliberately selected to resemble a use case where the embroidered structures can be used as wearable daily items. Nevertheless, the experimental results show the ability of the sensor to detect the presence of both water and glucose droplets, as well as changes to the concentration of glucose, all without the use of chemically specific substances being attached to the sensor. This indicates that the IDT-based fabric sensor has the potential to non-invasively monitor biomarkers. The addition of specific biomarker-selective chemicals enhances the sensitivity of glucose detection, and by selecting the correct chemical structures, other biomarkers could be identified and their levels monitored, leading to a full suite of non-invasive biomarker detection sensors.

## 4. Conclusions

In conclusion, this study unveils the potential of fabric-based IDT sensors as an innovative solution for non-invasive, continuous health monitoring, customizable to individual needs. Leveraging advanced embroidery techniques, the fabrication of flexible sensors on fabric swatches or directly into clothing items with gold-coated IDTs offers a novel approach. The demonstrated ability of these sensors to effectively and wirelessly detect water droplets and various glucose concentrations in water marks a significant advancement in health monitoring technology. This work demonstrates the feasibility of using an embroidered sensor that can be easily incorporated into daily items for molecule sensing without the need for direct electrical connections. It is envisioned that this sensor and method will eventually be used for sweat analysis. Additional functionalities such as sweat sampling will be added to the sensors toward this goal. In addition, the complex composition of sweat samples, including several molecules, will be investigated using the proposed platform. This study is the first important step toward this goal presenting the sensor structure, its fabrication and the wireless readout mechanism.

The embroidered IDT sensors offer unique advantages, including seamless integration into everyday clothing. This feature ensures a non-disruptive, user-friendly experience, aligning with the contemporary shift toward continuous, personalised health data capture. The study’s findings open avenues for further research and development in the realm of embroidered sensor technology, suggesting its potential for broader applications beyond glucose monitoring. While the current method of measuring the frequency shift is not designed for portable and wearable systems, advances in wireless sensing technology could enable a transition from lab-based measurements to a fully wearable suite of sensors, which could feasibly be used in any conceivable environment.

## Figures and Tables

**Table 1:** Comparison of selected sensors.

Reference	Sensitivity (Hz/(mg/dL))
[34]	$1.38\times 10^{2}$
[35]	$1.82\times 10^{2}$
[36]	$5.94\times 10^{1}$
[37]	$7.59\times 10^{1}$
[38]	$2.11\times 10^{4}$
[39]	$5.60\times 10^{6}$
[40]	$3.33\times 10^{3}$
This work	$1.7\times 10^{5}$

## Data Availability

The data presented in this study are available in this paper.
